# Cognate interactions: Extrafollicular IL-4 drives germinal-center reactions, a new role for an old cytokine

**DOI:** 10.1002/eji.201444825

**Published:** 2014-07-16

**Authors:** Kai-Michael Toellner

**Affiliations:** School of Immunity and Infection, Medical School IBR, University of BirminghamBirmingham, United Kingdom

**Keywords:** B cells, Germinal center, IL-4, IL-13, STAT6

## Abstract

Over the past 25 years it has become clear that B and T lymphocytes go through a range of interactions and migratory events when B cells differentiate to become high-affinity, antibody-secreting cells. This B-cell differentiation is associated with multiple sequential cognate interactions. In this issue of the *European Journal of Immunology*, Turqueti-Neves et al. [Eur. J. Immunol. 2014. 44: 2130–2138] show that IL-4, a cytokine well known as a regulator of Ig class switch recombination, has another as-yet-unappreciated role. The authors show that IL-4 produced by T-helper cells outside germinal centers has a major effect on the early stages of germinal-center B-cell differentiation. This Commentary will summarize their findings and relate them to what we know on the sequence of cognate interactions and migratory events B cells undergo during T-dependent immune responses.

See accompanying article by Turqueti-Neves et al.

B and T cells are members of the adaptive immune cell family and relations within the family are complex 1. To start with, Ag-induced B-cell differentiation alone is complicated: B cells not only proliferate, class switch their Ig genes in order to diversify function, and go through somatic hypermutation and selection events to increase Ab affinity, they also do this while migrating through a variety of different lymphoid microenvironments starting with the B-cell follicle and marginal zone, diving into the T zone, and moving back into the follicle again. On top of this involved series of events, B cells repeatedly interact with T cells, while the T cells themselves differentiate alongside B cells. Th cells polarize into different subspecialized populations with different effector functions; the definitions of these subpopulations, which subpopulations interact with B cells, and at which stage, are still subject to investigation and definition 2,3.

Activation by Ag causes B cells and T cells to meet for their first cognate interaction at the boundary between the T-zone and the B-cell follicles in secondary lymphoid organs such as spleen and lymph node 1,4,5. In B cells, this migration (Fig. 1) is guided by a balance of increased expression of the oxysterol receptor EBV-induced molecule 2 (Ebi2; gp183) 6 and the chemokine CCR7 7. While initially Ebi2 signals induced by the BCR may override CCR7-dependent B-cell migration toward the T-zone 6, eventually CCR7-dependent migration prevails and drives activated B cells toward the follicle — T-zone interphase 6,8. T cells primed on Ag-presenting DCs express CXCR5, the receptor for CXCL13 expressed throughout B-cell follicles, and this drives the movement of T cells toward B-cell areas 9,10. It is at the follicle — T-zone interphase that primed T cells can provide signals for cognate B-cell stimulation for the first time, that is CD40L 11 and/or T-cell cytokines that induce Ig class switch recombination 12. After cognate interaction between B cells and T cells, CCR7 ligand sensitivity is lost and B cells and T cells move back toward the outer boundaries of the follicles, a migration that is driven by a loss of CCR7 and prevailing signaling of Ebi2 6,13. Eventually, both cells end up in the interfollicular areas 5,8. Interfollicular areas in lymph nodes are located at the edges of the T-zone toward the subcapsular sinus (Fig. 1), or in spleens at the T-zone — red pulp bridging channels. These zones are special because cellular traffic, Ag entry, and prolonged interactions between DCs, activated B cells and T cells all take place here 5,8,14. Signals critical for GC development are exchanged in these sites 15. It is only after the loss of Ebi2 expression 6,16, and the induction of S1P2 17, that B cells assemble at the centre of the follicles to form GCs, the sites where hypermutation, affinity maturation, and further cognate interactions between B cells and T cells 18 take place (Fig. 1).

**Figure 1:**
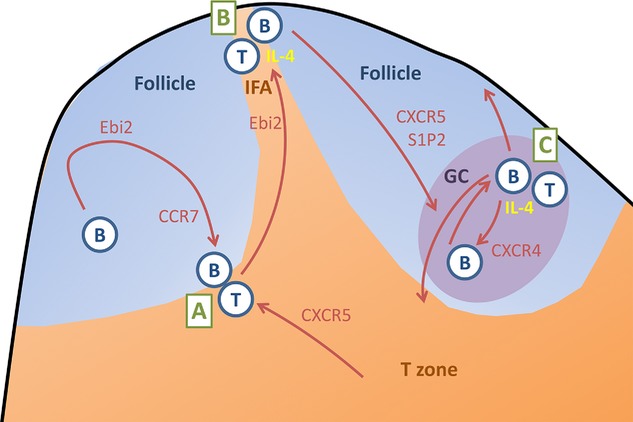
Cognate interactions: B and T lymphocytes, brothers and sisters moving hand in hand. (A) An initial interaction at the T–B interphase of secondary lymphoid organs is guided by the balance of sensitivity to Ebi2, CCR7, and CXCR5 ligands. CD40 ligation during cognate T cell–B cell interaction induces loss of CCR7 and increased Ebi2 sensitivity, followed by B-cell movement toward the interfollicular area (IFA) 6. (B) In interfollicular areas B cells and T cells may undergo prolonged interaction, probably exchanging IL-4 that may lead to the induction of Bcl6 and downregulation of Ebi2. (C) This finally leads to CXCR5- and S1P2-mediated clustering in the follicle centre, where B cells and T cells can differentiate as GC cells. Differentiation inside GCs involves further cognate T–B cell interactions leading to further selection and differentiation of GC B cells.

Th cells follow B cells along this journey of differentiation 5,19. The key regulator for T cells to migrate toward B cells is the expression of CXCR5, and CXCR5 is used as a marker for defining T follicular helper (Tfh) cells 10. Transcriptional polarization of Th cells is better described by the expression of polarizing transcription factors, and Bcl6, also expressed by B cells in the GC, turns out to be most useful transcription factor to define the population of T cells involved in activating B cells within follicles 19,20. Originally, the function of Tfh cells was presumed to solely be the selection of B cells in follicles, that is the GC; however, it is now clear that T cells can already polarize toward a Tfh-like phenotype before GCs are formed 21. The key cytokine produced by Tfh cells is IL-21, which has been shown to drive B cells toward a plasma cell phenotype 22,23. IL-4 and IL-13, are well known to polarize T cells toward the Th2 subset, but also act on B cells to induce class switch recombination 12, and have also been shown to be produced by Tfh cells in the GC environment 13,24.

In this issue of the *European Journal of Immunology*, Turqueti-Neves et al. 25 have revealed a new aspect to this story by showing how IL-4R signaling initiates the GC response. The authors started off by using helminth or lymphocytic choriomeningitis virus (LCMV) infections to induce Th2- or Th1-type immune responses. Both types of responses can result in the development of GCs. By using IL-4 and IL-13 double-deficient mice, the authors showed that within Th2 responses IL-4 receptor signals are important for GC formation 25. IL-13, being highly homologous to IL-4 with largely overlapping functions, is the other possible ligand and activator of signaling through the IL-4 receptor. GCs examined 12 days after infection with helminths or after immunization with ovalbumin in alum were considerably smaller in the absence of IL-4R signaling, confirming earlier observations by other groups 24,26. No major differences in GC B-cell death were seen without IL-4 or IL-13, which might have been expected if vital selection signals from Tfh cells were missing. However, a slight increase in B-cell proliferation in IL-4/13-deficient GCs compared with that in WT GCs indicated that things were not entirely in the same balance as in WT GCs: increased B-cell proliferation in the absence of IL-4R signaling could indicate reduced input, increased output of cells leaving the GC, or changes in B-cell apoptosis too subtle to detect with the methods used in this study.

To test whether IL-4/IL-13 signaling inside the GC is important for maintenance of GC size, mixed BM chimeras were produced where one half of the T cells were IL-4/13 double-deficient and able to express CXCR5, while the other half was CXCR5-deficient and competent to produce IL-4/13. In this system, Tfh cells should be IL-4/13-deficient, while Th cells competent to produce IL-4/13 would be excluded from the GC because they are CXCR5-deficient. The latter group, however, would be able to produce IL-4/13 during the extrafollicular phase of the response. Surprisingly, in this model, GCs developed to a normal size, containing normal numbers of Tfh cells 25. Also, normal levels of Ig class switching to IgG1 ensued, demonstrating that much Ig class switching is induced early on during the extrafollicular phase of an Ab response. To test whether extrafollicular Th2 cells may induce GC differentiation in this response, classical Th2-polarized cells that did not express CXCR5 were isolated from helminth-infected mice and added to an established GC response in IL-4/13-deficient animals. Five days later, GCs in these animals were considerably larger, while the transferred Th2 cells were still not expressing CXCR5 25. While this last experiment does not exclude the possibility of transient development of Tfh cells from the transferred Th2 cells, it adds to the argument that extrafollicular cytokines produced by Th2 cells are important in directing what happens inside the follicle.

Whether the effects from IL-4/13 signaling are B-cell-intrinsic was tested in BM chimeras that contained a mix of lymphocytes that either were or were not able to produce STAT6 25, a transcription factor that is downstream of IL-4R signaling 27. The authors show that IL-4R signaling in B cells is essential for GC B-cell differentiation, while it is dispensable for Tfh-cell development. Although the experiments show that extrafollicular IL-4/13 signals are important for GC differentiation 25, the question as to whether gene expression in the GC B cells that do develop was changed in STAT6-deficient mice, and what genes might be involved, is still open. A screening of a small number of GC-related genes gave an exciting hint: while some GC-associated genes such as AID, Bcl6, and XBP1 were reduced in the absence of STAT6 signaling, interestingly, mRNA coding for Ebi2, the receptor that drives B cells away from the follicle centre, was 30-fold increased 25.

There are several interesting observations in this study. First, much of the Ig class switching that is occurring during the early Ab response is induced during the extrafollicular phase — outside GCs. This is not too surprising, as it has been described by us and others that interaction with Th cells at this early phase induces IgG class switching T-dependent signals 4,12,28,29. What Turqueni-Neves et al. 25 did not control for was what happens if IL-4/13 were not available during the extrafollicular phase of B-cell activation. Had they done so, they may have found another surprise, which is that during this early phase IL-4/13 signals are not essential to regulate Th2-polarized IgG1 class switching 26,30. While IL-4R signals are essential for IgE class switching, the nature of the signals inducing extrafollicular Th2-type class switching in the absence of IL-4/13 are still unknown.

Second, in Th2 responses, extrafollicular Th2 cells and not Tfh cells induce the differentiation of GC B cells. While the authors qualify their finding by stating that it cannot be excluded that the transferred Th2 cells at some stage were entering follicles and expressing CXCR5 25, the experiments are a strong indication that committed Th2 cells in the extrafollicular phase stimulate pre-GC B cells. Using the Tfh-specific transcription factor Bcl6 as a marker for Tfh-cell polarization, others have demonstrated recently that Tfh cells appear during extrafollicular B-cell activation where they provide IL-21 21,31. So are the early helpers Th2 cells or Tfh cells? This may be a matter of the particular nature of the immunogen, or just of definition. The present study used CXCR5 expression as a marker for extrafollicular cells 25. Extrafollicular cognate interactions should be possible without CXCR5 expression on Th cells, but during these early stages of extrafollicular T-cell differentiation, commitment to any T-cell polarization stage may still be vague, and T cells may still have bispecific potential or be able to interconvert between types 2.

Third, loss of the IL-4R signals on B cells during extrafollicular differentiation results in GC B cells that express fewer GC-associated genes. This is accompanied by considerably higher levels of Ebi2 message in B cells, thereby maintaining B cells in interfollicular areas as opposed to traffic to follicular centers 6,8. In these interfollicular areas Ag enters, and B cells are in close vicinity to activated T cells and Ag-activated DCs 14. In the spleen, interfollicular areas are also the places where extrafollicular plasmablast differentiation begins 1. Interfollicular areas have been shown to accommodate prolonged cognate interactions that signal B cells to take the GC pathway 15. IL-4 has been demonstrated in these interfollicular areas 24 and it may be that one function of IL-4 produced in this area is to induce loss of Ebi2 expression in B cells, leading to GC differentiation (Fig. 1).

How would IL-4 downregulate Ebi2? STAT6 is a transcriptional activator. The transcriptional repressor Bcl6 has been shown to target STAT6 recognition sites and may repress Ebi2 this way 8,27. While initial BCR signaling itself tends to inhibit Bcl6 expression 29, Bcl6 has been shown to be induced during cognate interactions in the interfollicular areas 15. Possibly IL-4 itself is the stimulus that induces Bcl6 in this site. Bcl6 has been identified at least once as a potential target of STAT6 32. This could make a nice negative feedback loop, where Bcl6 would inhibit STAT6 action, after STAT6 has initiated the transcription of Bcl6.

So what then is the high production of IL-4 in Tfh cells within GCs 13,24 good for? It is of course possible that the IL-4 produced by Tfh cells in the GC has a similar function, signaling B cells to stay inside the GC. The lack of an effect of absent IL-4/13 from Tfh cells may be too minor and compensated by increased B-cell proliferation. IL-4 may have different functions; IL-4 deficiency has been shown to result in reduced affinity maturation, which would be easily explained with GC-intrinsic effects on B-cell selection 24. We also do not know which signals induce B cells to exit the GC. IL-21, produced by Tfh cells, has been shown to affect plasma cell differentiation 22. IL-4 in the GC may have other functions, for example reinforcing Th2 type Ig class switching recombination during the GC phase of the response 26, or induction of memory B-cell formation. The work of Turqueti-Neves et al. however shows that extrafollicular IL-4, apart from its known immediate effects inducing B-cell class switch recombination, also has important downstream effects initiating the differentiation of B cells toward the follicular GC pathway.

## Abbreviations

Ebi2: EBV-induced molecule 2
Tfh: T follicular helper
